# Nutrient intakes of rural Tibetan mothers: a cross-sectional survey

**DOI:** 10.1186/1471-2458-10-801

**Published:** 2010-12-31

**Authors:** Zhenjie Wang, Shaonong Dang, Hong Yan

**Affiliations:** 1 Department of Epidemiology and Health Statistics, School of Public Health, Xi'an Jiaotong University College of Medicine, Xi'an, Shaanxi 710061, P.R. China; 2 Department of Preventive Medicine, Graduate School of Medical Sciences, Kyushu University, Maidashi 3-1-1, Higashi-ku, Fukuoka 812-8582, Japan

## Abstract

**Background:**

Tibetan food intake is influenced by the region's high altitude and unique culture. Few published studies of nutrient intakes among Tibetan women are available. The present study of Tibetan mothers with young children explores dietary patterns, nutrient intakes, and differences between socio-demographic groups.

**Methods:**

A cross-sectional survey of 386 women with a child aged less than 24 months was conducted in rural areas surrounding Lhasa, Tibet. All participants were recruited using simple random sampling and were interviewed face-to-face by trained investigators. Dietary information was collected via a food frequency questionnaire. Nutrient intakes were calculated using food composition tables. Non-parametric tests were used to compare nutrient intakes according to socio-demographic variables, and to compare results with the *2002 Chinese National Nutrition and Health Survey *(2002 NNHS) and dietary reference intakes (DRIs).

**Results:**

Median intakes of energy (*p *< 0.001), protein (*p *< 0.001), fat (*p *< 0.001), vitamin A (*p *< 0.001), vitamin B1 (*p *< 0.001), vitamin B2 (*p *< 0.001), vitamin C (*p *< 0.001), and vitamin E (*p *< 0.001) were lower than the average levels reported in 2002 NNHS. The median intakes of calcium (517 mg/d, *p *< 0.001), iron (35 mg/d, *p *< 0.001), and zinc (17.3 mg/d, *p *< 0.001) were higher than the average levels in 2002 NNHS. The highest education subgroup had significantly higher intakes of vitamins A and C than the lowest education subgroup.

**Conclusion:**

Although the diet of Tibetan mothers with young children has been partially influenced by other factors, their dietary patterns are still mostly composed of Tibetan traditional foods. Compared with the 2002 NNHS, Tibetan women with young children appear to have insufficient intakes of many nutrients, which will affect their nutritional status.

## Background

According to the *2002 Chinese National Nutrition and Health Survey *(2002 NNHS) [1], the prevalence of anemia in China was over 25% (Dallman et al. method) among women from rural areas. A hospital-based survey showed the prevalence of anemia was variously 70.0% (CDC method), 77.9% (Dirren et al. method), and 41.3% (Dallman et al. method) among Tibetan women in Lhasa, Tibet [2]. Furthermore, the 2002 NNHS results also showed that 8.3% of women from rural areas were malnourished and suggested that these women lacked basic knowledge of nutrition and health [1]. However, in the 2002 NNHS, only 0.8% of the women sampled were Tibetan; therefore, the nutritional status of Tibetan women could not be specifically described.

There have only been a few studies describing the nutrient intakes of Tibetans [3,4]. Moreover, these studies have not provided a sufficient explanation of the dietary intake circumstances of Tibetan mothers. In 2008, we began a research project in Lhasa, Tibet, that mainly focuses on two points: first, the health status of children aged 0-24 months and the behaviors and attitudes of their mothers towards feeding; and second, the mothers' dietary and nutrient intakes. The survey was supported by the National Natural Science Foundation of China and the China Medical Board of New York, Inc. In the current analysis, we describe Tibetan mothers' nutritional status, and compare the differences between nutrient intakes of Tibetan women and the results for lactating mothers in the 2002 NNHS. We also analyze the associations between nutrient intake and socio-demographic characteristics.

## Methods

### Setting and survey design

Tibet is located on the highest plateau of the world, with 86% of the region at an altitude of at least 4000 meters. Most Tibetans live at altitudes between 2500 and 4500 meters [5], and the main economical activities are farming and animal husbandry [6]. The 2003 population census indicated that there were approximately 2.59 million inhabitants in Tibet, with 96% being of Tibetan nationality [6]. The rural population accounted for approximately 85% of the total population [7]. The study was conducted in rural areas surrounding Lhasa, the capital of Tibet, which is located on the Qinghai-Tibet Plateau. The mean altitude of Lhasa is 3685 meters above sea level.

A cross-sectional survey was designed to document the health status of children aged under 24 months and their mothers' dietary and nutrient intakes. Participants were selected using simple random sampling and were interviewed face-to-face by trained professional interviewers between May and August 2008. The study was approved by the Ethics Review Committee, Xi'an Jiaotong University College of Medicine. All participants signed a consent form before completing the interview.

### Sample size

Since the purpose of our study was to measure the health status of children aged under 24 months and the dietary intakes of mothers, we first calculated the sample size based on the rates of diarrhea (67.2%) and upper respiratory infection (67.3%) reported in the 2002 NNHS and the rate of breastfeeding (85%) in the Development of Chinese Children (2001-2010). The sample size equations were as follows: *α *= 0.05, *δ *= |*p*-*π*| = 0.05, followed by n=μα22π(1−π)δ2. The required sample sizes were 339, 338, and 196, respectively. We chose the maximum sample size (339), and expected a 20% no-response rate. The final sample size was 406. Furthermore, there was a lack of precise statistics for the nutrient intakes of Tibetan mothers with young children. However, a sample size of 382 was used for lactating mothers' nutrient intakes in the 2002 NNHS [1]; we used a sample size of 406 to describe Tibetan mothers' nutritional status. We selected the participants using simple random sampling based on the list of children who were under 24 months old and their mothers in the study area. A total of 386 participants completed the study (95.1%).

### Socio-demographics and anthropometry

Standardized interview questions were used to collect socio-demographic information, including age, years of education, maternal occupation, and family size, as described in previous studies in Tibet [8,9]. Subjects were asked to remove all heavy clothes and shoes and undo hair styles and accessories in a preparation area. Next, trained staff weighed the subjects on a calibrated electronic scale (Tanita HD-305, Tanita (Shanghai) Trading Co., Ltd.) and recorded the value to the nearest 0.1 kilogram. Standing height was measured using a height-measuring tape (LD-SG01, Ningbo Land cooperation) suspended from the wall. The subjects were asked to stand erect with their shoulders level, hands at their sides, thighs together, and heels comfortably together. Subjects also kept the upper back, buttocks, and heels in contact with the wall and the head aligned in the Frankfort plane during the height measurement. The height values were recorded to the nearest 0.1 centimeter. All anthropometric measurements were taken by a single trained staff member. Body mass index (BMI) was calculated as weight (in kilograms) divided by height (in meters) squared. Women were classified into BMI less than 18.5 kg/m^2^, 18.5-24.9 kg/m^2^, 25-29.9 kg/m^2^, or over 30 kg/m^2 ^[10].

### Dietary survey

The mothers' nutritional status was determined from the dietary data collected using a food-frequency questionnaire (FFQ). The FFQ has adequate reproducibility and validity for assessing dietary intake in rural China [11,12]. Since this survey was conducted in Tibet, we added some Tibetan foods to the questionnaire, such as Zanba, Tibetan milk tea, and Tibetan salt cream tea. Before being used in the study, the FFQ was revised twice with the assistance of local nutrition staff. After each revision, the FFQ was re-tested to determine whether the items fitted local daily food consumption patterns. The final version of the FFQ included 92 items, covering cereals, meat, vegetables, fruits, egg products, nuts, fish, beverages, alcoholic beverages, and snacks. Typical dishes were displayed in the booklet, along with average portion sizes [13,14]. Options for the serving size were 0.5, 1, 1.5, or 2, with the displayed size as reference (average size) for most of the food items. More detailed options were offered for portion sizes of Tibetan foods. Participants were asked how often, on average, they consumed each food item during the past 12 months.

Intakes were calculated for energy, protein, fat, carbohydrate, vitamin A, vitamin B1, vitamin C, vitamin E, calcium, iron, and zinc. Nutrients were calculated according to the 2002 and 2004 China Food Composition Tables [15,16]. Food items were divided into six different subgroups, following previous experience of nutritional investigations and dietary habits in the rural area of western China [11,12].

### Nutrient reference values

Nutrient reference values were based on national standards [16]. Since these were rural participants, we used Chinese DRIs (dietary reference intakes) with moderate PAL (physical activity level). Reference values were RNI (recommended nutrient intake) or AI (adequate intake) for lactating mothers. These recommended values are: energy 11721 kJ, protein 90 g, fat at 20-30% of total energy (63.4-95.0 g), and carbohydrate at 55-65% of total energy (379.2-448.2 g). We chose the median of the range for fat (79 g) and carbohydrate (414 g) as the recommended value. Other nutrient reference values were as follows: vitamin A 1200 μg (retinol equivalents), vitamin B1 1.8 mg, vitamin B2 1.7 mg, vitamin C 130 mg, vitamin E 14 mg, calcium 1200 mg, iron 25 mg, and zinc 21.5 mg. In the present analysis, we compared our results with the daily nutrient intakes of lactating mothers in the 2002 NNHS. In that survey, dietary intake data were collected using 24-hour recall over 3 consecutive days [1]. The nutrient intakes of lactating mothers in the 2002 NNHS were as follows: energy 10036 kJ, protein 70 g, fat 77 g, carbohydrate 355 g, vitamin A 524 μg, vitamin B1 1.1 mg, vitamin B2 0.8 mg, vitamin C 96 mg, vitamin E 37 mg, calcium 385 mg, iron 24 mg, and zinc 11.6 mg.

### Quality control

The interviewers (physicians from the Department of Public Health, School of Medicine, Tibet University) were trained to standardize administration of the questionnaire and anthropometric measurements. They were trained in the field for at least 1 week prior to commencing the surveys. A pilot survey was performed before the formal survey, with all data for analysis being from the formal survey. Two investigation teams were established, each consisting of four members and a supervisor. At least two members were Tibetan and were able to communicate in both Tibetan native language and Chinese. During the survey, a data checking system was employed, which involved all interviewers checking their own data, each interviewer checking data with another interviewer, and data checking by supervisors. Subjects were re-interviewed when answers to key questions or missing values were identified. The accurate age of the child was based on the Permanent Residence Registration and/or Record of Scheming Immunization documents, where the birth date is recorded. The Tibetan lunar calendar dates were converted to Gregorian calendar dates.

### Statistical analysis

A database was established by using Epi Data version 3.1 (The EpiData Association Odense, Denmark), and double data entry was performed. We used the Shapiro-Wilk test to determine whether nutrient intakes had a normal distribution. The data for nutrient intakes had a non-normal distribution; therefore, median values were used to describe continuous variables. We calculated the consumption frequency of all foods, and arranged the five most frequently consumed foods from each food subgroup in descending order. We used the Wilcoxon signed rank test to determine whether our data were significantly different from 2002 NNHS values. To compare the differences between different socio-demographic groups, we used the Kruskal-Wallis test. To compare the differences between two subgroups within each socio-demographic group, we used the Wilcoxon two-sample test (adjusted *P *< 0.017). Statistical significance was determined at *P *< 0.05 (two-tailed tests). SAS version 9.2 (SAS Institute Inc., Cary, NC, United States) was used for all analyses.

## Results

### Socio-demographic characteristics

All 386 participants were native Tibetans. Approximately 89% of the children had been breastfed, and 49% were still breastfeeding during the survey (data not shown). The mean age of the mothers was 28.5 years. Approximately 20% of the participants had completed 9 years of basic education. The quartile range for family size was 4-7 people in one household. Most earned a living by farming and raising animals only. There were no women classified as obese (Table 1).

**Table 1:** Socio-demographic characteristics of the subjects

Characteristic	Outcomes n (%)
Maternal age, y (n = 385)	
< 20.0	6 (1.6)
20-23.9	86 (22.3)
24-29.9	168 (43.6)
≥30	125 (32.5)
Educational years (n = 385)	
< 1	121 (31.4)
1-8	183 (47.5)
≥ 9	81 (21.0)
Maternal occupation (n = 384)	
Farming and animal husbandry only	277 (72.1)
Others	107 (27.9)
Family size (n = 386)	Median (25th%-75th%)
	5 (4-7)
Number of children in each household (n = 386)	
1	225 (58.3)
2	140 (36.3)
≥3	21 (5.4)
Body mass index, kg/m^2 ^(n = 361)	
< 18.5	37 (10.3)
18.5-24.9	294 (81.4)
25-29.9	30 (8.3)

### Dietary intake of Tibetan mothers

In this survey, staple foods provided 64.1% of total energy, 55.2% of total protein, 27.3% of total fat, 75.9% of total carbohydrate, 45.4% of total calcium, 71.2% of total iron, and 74.6% of total zinc. Meat, poultry, and fish provided 13.0% of total protein and 10.6% of total fat. Eggs, milk, and dried legumes provided 7.7% of total calcium (Table 2). On average 73%, 11%, and 24% of dietary energy originated from carbohydrates, proteins, and fat, respectively (data not shown).

**Table 2:** Median nutrient intake (percentage of total intake) derived from different dietary food groups

	Energy (kJ/d)	Protein (g/d)	Fat (g/d)	Carbohydrate (g/d)	Vitamin A (μg/d)*	Vitamin B1 (mg/d)	Vitamin B2 (mg/d)	Vitamin C (mg/d)	Vitamin E (mg/d)	Calcium (mg/d)	Iron (mg/d)	Zinc (mg/d)
Staple												
Wheat products	1587.3 (18.1)	10.7 (18.6)	3.4 (6.0)	78.6 (22.0)	4.4 (1.8)	0.02 (2.3)	0.04 (6.3)	/^†^	0.8 (6.8)	32.1 (6.2)	2.5 (7.1)	1.01 (5.8)
Rice products	1336.6 (15.2)	7.3 (12.7)	0.9 (1.7)	71.0 (19.9)	/^†^	0.05 (5.7)	0.08 (12.5)	/^†^	0.3 (2.1)	19.8 (3.8)	3.4 (9.7)	2.32 (13.4)
Tibetan naked barley	2418.3 (27.6)	12.9 (22.4)	9.9 (17.4)	117.7 (33.0)	/^†^	0.51 (58.0)	0.21 (32.8)	/^†^	2.2 (18.2)	185.3 (35.9)	16.6 (47.8)	8.38 (48.4)
Meat, poultry, and fish												
Meat	332.4 (3.8)	6.6 (11.5)	5.5 (9.7)	0.9 (0.3)	4.4 (1.8)	0.04 (4.6)	0.04 (6.3)	< 0.01	0.2 (1.7)	6.6 (1.3)	1.0 (2.8)	1.32 (7.6)
Poultry and fish	14.7 (0.2)	0.4 (0.7)	0.2 (0.3)	0.02 (0.01)	0.9 (0.4)	< 0.01	< 0.01	/^†^	0.01 (0.1)	0.2 (0.04)	0.03 (0.1)	0.02 (0.1)
Eggs, milk, and dried legumes												
Eggs and milk	84.1 (1.0)	1.2 (2.2)	1.0 (1.8)	1.4 (0.4)	17.3 (7.1)	0.01 (1.1)	0.03 (4.7)	0.2 (0.3)	0.1 (1.0)	24.2 (4.7)	0.2 (0.5)	0.14 (0.8)
Dried legumes	17.2 (0.2)	0.3 (0.5)	0.3 (0.5)	0.2 (0.1)	< 0.01	< 0.01	/^†^	/^†^	0.2 (1.5)	8.6 (1.7)	0.1 (0.3)	0.05 (0.3)
Vegetables, tubers, and starches	263.3 (3.0)	2.5 (4.4)	0.3 (0.5)	14.0 (3.9)	55.4 (22.8)	0.07 (8.0)	0.07 (10.9)	40.4 (59.5)	0.9 (7.3)	35.0 (6.8)	1.5 (4.3)	0.52 (3.0)
Fruits and nuts	396.8 (4.5)	2.4 (4.1)	4.6 (8.1)	8.9 (2.5)	28.5 (11.7)	0.07 (8.0)	0.05 (7.8)	6.1 (8.9)	3.0 (24.9)	14.1 (2.7)	/^†^	/^†^
Drinks and snacks												
Drinks	593.2 (6.8)	3.4 (5.9)	11.5 (20.3)	6.0 (1.7)	64.8 (26.7)	0.04 (4.6)	0.03 (4.7)	2.3 (3.4)	0.3 (2.4)	100.5 (19.5)	1.6 (4.5)	0.68 (3.9)
Snacks	650.9 (7.4)	1.9 (3.3)	3.3 (5.7)	25.9 (7.3)	/^†^	0.04 (4.6)	0.03 (4.7)	3.6 (5.3)	0.3 (2.9)	11.7 (2.3)	1.5 (4.4)	0.26 (1.5)

* Retinol equivalent

† Value was below the limit of detection or trace amount

The most commonly consumed foods were Tibetan foods such as salt cream tea, Zanba, Tibetan milk tea, yak meat, Tibetan noodles, and salted boiled potato (exceptions were rice and noodles). These are traditional foods that are either from unique crops or are made using a local cooking style. In particular, the frequencies of consumption of Tibetan salt cream tea and Zanba were approximately once per day. Other traditional foods, such as Tibetan noodles, yak, and Tibetan milk tea, were consumed 2-4 times per week. In contrast, most other foods were consumed 1-3 times per month: eggs, milk, and dried legumes; vegetables, tubers, and starches; and fruits and nuts (Table 3).

**Table 3:** The five most frequently consumed foods from the six dietary food groups *

Order of consumption frequency	Staple	Meat, poultry and fish	Eggs, milk and dried legumes	Vegetables, tubers and starches	Fruits and nuts	Drinks and snacks
First	Rice (7)	Yak (5)	Egg (3)	Potato (5)	Sunflower seeds (5)	Tibetan salt cream tea (7)
Second	Zanba (6)	Pork (5)	Yogurt (3)	Shallot (5)	Apple (4)	Green Tea (7)
Third	Tibetan noodles (5)	Beef (4)	Milk (3)	Chinese cabbage (4)	Orange (3)	Tibetan milk tea (5)
Fourth	Wheat noodles (5)	Chicken (2)	Soybean soft curd (2)	Hot pepper (4)	Banana (3)	Potato salty boiled (4)
Fifth	Wheat bun (4)	/^†^	/^†^	Tomato (4)	Watermelon (2)	Mungbean starch jelly (4)

*Categories are: 7 (once per day), 6 (5-6 times per week), 5 (2-4 times per week), 4 (once per week), 3 (1-3 times per month), 2 (less than once per month), 1 (almost never)

† Other foods consumed less than one time per month

In this survey, we calculated energy, macronutrients, and micronutrients derived from the top five foods in the six dietary food groups (Table 3). These foods provided 74.7% of total energy, 70.2% of total fat, 75.9% of total protein, and 73.7% of total carbohydrate, as well as over 70% of the overall intake of other nutrients (data not shown).

### Comparison of nutrient intake with the 2002 NNHS and nutrient reference values

Intake of carbohydrate was similar to the 2002 NNHS, whereas intakes of calcium, iron, and zinc were higher than those reported in the 2002 NNHS. The majority of the iron was obtained from Zanba, rather than from the meat, poultry, and fish food subgroup (data not shown). Intakes of other nutrients were lower than the 2002 NNHS (Table 4).

**Table 4:** Energy and nutrient intakes in Tibetan mothers compared with the 2002 NNHS

	Median of total	RNI/AI^†^	(%)^‡^	2002 NNHS^†^	*P*^§^
Energy (kJ/d)	8778	11721	74.9	10036	< 0.001
Protein (g/d)	58	90	64.4	70	< 0.001
Fat (g/d)	57	79	72.2	77	< 0.001
Carbohydrate (g/d)	357	414	86.2	355	0.187
Vitamin A (μg/d)*	243	1200	20.3	524	< 0.001
Vitamin B1 (mg/d)	0.9	1.8	50.0	1.1	< 0.001
Vitamin B2 (mg/d)	0.6	1.7	35.3	0.8	< 0.001
Vitamin C (mg/d)	68	130	52.3	96	< 0.001
Vitamin E (mg/d)	12	14	85.7	37	< 0.001
Calcium (mg/d)	517	1200	43.1	385	< 0.001
Iron (mg/d)	35	25	140.0	24	< 0.001
Zinc (mg/d)	17.3	21.5	80.5	11.6	< 0.001

* Retinol equivalent

^†^RNI: recommended nutrient intake of lactating mothers; AI: adequate intake of lactating mothers; 2002 NNHS: mean intake per day of lactating mothers

^‡ ^Percent of RNI or AI

^§^Difference between median intake and average intake of lactating mothers in the 2002 NNHS using the Wilcoxon signed rank test (*P *< 0.05)

Energy, fat, carbohydrate, vitamin E, and zinc intakes were close to the nutrient reference values; however, iron intake was higher than the reference values. Other nutrients had low intakes (approximately 70% of the reference values) (Table 4). When nutrients were compared across socio-demographic groups, there were significant differences in nutrient intakes according to years of education (Table 5). The highest education subgroup had significantly higher intakes of vitamin A and vitamin C compared with the lowest education subgroup. There were no other significant differences between subgroups.

**Table 5:** Median values of nutrient intakes (percentages of nutrient reference values) by socio-demographic category

	Energy (kJ/d)	Protein (g/d)	Fat (g/d)	Carbohydrate (g/d)	Vitamin A (μg/d) ^‡^	Vitamin B1 (mg/d)	Vitamin B2 (mg/d)	Vitamin C (mg/d)	Vitamin E (mg/d)	Calcium (mg/d)	Iron (mg/d)	Zinc (mg/d)
Age, y*												
≤ 23.9	8682 (90.2)	58 (82.8)	59 (92.2)	341 (98.8)	237 (33.9)	0.9 (70.0)	0.7 (57.5)	75 (75.0)	12 (85.7)	516 (64.5)	34 (171.4)	17.4 (151.3)
24-29.9	9100 (94.5)	58 (82.8)	59 (92.2)	377 (109.3)	270 (38.6)	0.9 (70.0)	0.6 (51.7)	71 (71.0)	12 (85.7)	521 (65.1)	35 (175.7)	17.1 (149.0)
≥30	8447 (87.7)	55 (78.6)	52 (81.3)	351 (101.7)	224 (32.0)	0.9 (70.0)	0.6 (51.7)	58 (58.0)	11 (78.6)	511 (63.9)	35 (175.7)	17.6 (153.1)
Educational years^†^												
< 1	8138 (84.5)	53 (75.7)	53 (82.2)	326 (94.4)	213^b^ (30.4)	0.8 (63.1)	0.6 (51.7)	60^a^ (60.0)	11 (78.6)	482 (60.3)	33 (166.3)	17.3 (150.7)
1-8	9151 (95.1)	60 (85.4)	59 (92.2)	373 (108.2)	244 (34.9)	0.9 (70.0)	0.7 (57.5)	71 (71.0)	12 (85.7)	546 (68.2)	36 (179.0)	17.1 (149.0)
≥ 9	9218 (95.8)	61 (87.3)	62 (97.3)	375 (108.6)	293 (41.8)	0.9 (69.2)	0.6 (52.5)	84 (83.7)	12 (85.7)	511 (63.9)	33 (166.2)	17.6 (153.4)
Maternal occupation												
Farming and animal husbandry only	8669 (90.1)	57 (82.1)	56 (87.4)	360 (104.3)	235 (33.6)	0.9 (66.9)	0.6 (53.3)	68 (67.7)	12 (85.7)	515 (64.4)	34 (171.4)	17.3 (150.7)
Others	9180 (95.3)	60 (86.4)	60 (94.3)	354 (102.5)	279 (39.9)	0.9 (69.2)	0.6 (50.8)	69 (69.0)	12 (85.7)	523 (65.3)	35 (175.7)	17.3 (150.7)
Family size												
≤ 3	8414 (87.4)	53 (75.4)	52 (81.7)	346 (100.3)	216 (30.9)	0.9 (69.2)	0.6 (50.0)	58 (57.6)	10 (71.4)	513 (64.3)	35 (173.0)	16.7 (144.8)
4-7	8874 (92.2)	58 (83.0)	59 (92.0)	360 (104.3)	248 (35.5)	0.9 (66.2)	0.7 (55.0)	69 (69.2)	12 (85.7)	527 (65.8)	35 (176.8)	17.6 (153.1)
≥ 8	8514 (88.4)	58 (83.4)	51 (79.8)	362 (105.1)	231 (33.0)	0.9 (67.7)	0.6 (52.5)	82 (82.0)	12 (85.7)	496 (62.0)	30 (151.4)	15.8 (137.0)
BMI, kg/m^2^												
< 18.5	8786 (91.3)	58 (82.6)	58 (90.4)	371 (107.4)	282 (40.3)	0.9 (72.3)	0.6 (53.3)	80 (80.3)	13 (92.9)	539 (67.3)	35 (176.8)	18.0 (156.3)
18.5-24.9	8594 (89.3)	57 (81.6)	55 (86.8)	350 (101.4)	235 (33.6)	0.9 (66.2)	0.6 (52.5)	64 (64.0)	11 (78.6)	514 (64.3)	33 (166.7)	17.3 (150.7)
25-29.9	8318 (86.4)	56 (79.5)	52 (81.3)	348 (100.9)	171 (24.5)	0.8 (61.5)	0.6 (53.3)	73 (72.8)	11 (78.6)	494 (61.8)	36 (181.0)	17.2 (149.5)

* Age categories of < 20.0 and 20-23.9 years are presented as < 23.9

^†^Significant differences in intakes of protein, vitamin A, and vitamin C according to years of education (*P *< 0.05); ^‡ ^Retinol equivalent

a significant difference in vitamin C intake for < 1 versus 1-8 years of education using the Wilcoxon two-sample test (*P *< 0.017)

b significant difference in vitamin A intake for < 1 versus ≥ 9 years of education using the Wilcoxon two-sample test (*P *< 0.017)

## Discussion

Our current survey provided detailed data on Tibetan mothers' dietary patterns and daily nutrient intakes and showed that the diets of these mothers with young children are mainly derived from staple foods (cereals) and may not be nutritionally balanced. Our findings suggest that approximately 25% of the foods taken frequently were traditional Tibetan foods; however, interestingly enough, the results also show that traditional Han foods, such as rice, wheat noodles, and buns, were also frequently consumed in the Tibetan diet. Our findings provide evidence that Tibetan dietary patterns mainly reflect consumption of cereals [4]. Vegetables and fruits are eaten less frequently. This indicates that rural Tibetan mothers' diets consist predominantly of staple and traditional Tibetan foods, which may be attributed to their traditional Tibetan culture. Similar dietary patterns have been observed among other minority nationalities in China [17]. Compared with previous findings [4], we recorded somewhat lower energy and protein intake per day.

An interesting finding was that the intakes of iron and zinc were significantly higher than the 2002 NNHS, and the intake of iron was notably higher than the nutrient reference values, mainly due to the consumption of Zanba (which consists of Tibetan naked barley, Tibet salt cream tea, and Tibetan cream). Zanba has higher levels of iron (13.9 mg/100 g) and zinc (9.55 mg/100 g) than regular wheat flour, which contains 3.5 mg/100 g of iron and 1.64 mg/100 g of zinc [15]. There have been few studies on the bioavailabilities of iron and zinc from Zanba. Other research on the bioavailablities of iron and zinc may provide some clues. Heme iron is estimated to have a uniform absorption of 15-35%. However, non-heme iron has much lower bioavailability than heme iron [18-20], with an absorption rate of 2.8% from the consumption of cereals, pulses, and vegetables [21]. The bioavailability of zinc is influenced by the degree to which flour is processed: almost 50% more zinc is absorbed from a serving of whole-wheat bread compared with a serving of white bread [22]. According to our survey, iron and zinc were mainly derived from plant foods and their absorption may be limited, which might explain the higher prevalence of anemia observed among Tibetan women in other studies [2]. Intakes of calcium were higher than the average level in the 2002 NNHS, which might be due to the traditional Tibetan milk tea (black tea, milk, sugar) that is consumed every day.

Although the diets of these women provided close to sufficient intakes of calcium, iron, and zinc, intakes of most other nutrients (especially vitamins) were lower than the average level in the 2002 NNHS. Previous research suggested that dietary diversity was correlated with nutrient intakes [23,24]. Therefore, the main reason for low intakes of vitamins may be the lower frequency of consumption of vegetables, tubers, and starches and fruits and nuts. These foods accounted for 33 items in our FFQ but only seven items had a frequency of at least once per week; other foods in these subgroups were consumed less frequently. Intakes of protein and fat were significantly lower than the 2002 NNHS. This may be because, among the 21 items in the two dietary food groups (meat, poultry, and fish and eggs, milk, and dried legumes), only two foods (yak and pork) were consumed at least once per week.

Several studies have suggested associations among socioeconomic groups, demographic characteristics, culture, lifestyles, and dietary habits [17,25-32]. Factors such as gender, age, education level, and physical activity appear to affect eating habits. Women who are wealthier and/or highly educated individuals appear to have healthier diets [25-27]. In our study, we also found that socio-demographic factors including education were associated with nutrient intakes of Tibetan mothers with young children. Our results suggest that the education status of the mother may influence her dietary intake. It is possible that well-educated mothers are more able to eat a balanced diet than those with low levels of education. A potential reason for this is that mothers who have a greater number of years of education have more opportunities to accumulate nutritional knowledge in school and may, therefore, pay more attention to their dietary intakes.

There are some limitations of the present study. First, although the cross-sectional design of the survey yields information about the nutritional status of Tibetan mothers with children under 2 years of age, this design does not provide direct evidence of the causality [33]. Therefore, this characteristic should be noted when the results are used in other research. Second, the selection of a moderate PAL as a reference could introduce bias because we have not measured the PALs of the women directly. However, all of the women had children and/or were breastfeeding their infants (approximately 50%), which made it impossible for them to do hard physical labor such as farming all day. We believe that a moderate PAL would be appropriate for them. Third, we were unable to obtain blood samples from the participants; thus, we did not have access to clinical biomarkers of their nutritional status (such as serum hemoglobin). The measurement of biomarkers for nutritional status may help us to understand whether unbalanced dietary patterns are leading to poor nutritional status among rural Tibetan mothers with young children. For example, 10.3% of women in this sample were underweight, which was slightly higher than the 3.1% of mothers with children under 2 years old who were underweight in the 2002 NNHS [1]. Although there are some limitations to our present study, it nevertheless provides some valuable information on the dietary patterns and nutrient intakes of Tibetan mothers with young children.

## Conclusions

Rural Tibetan mothers with young children were shown to have fairly monotonous dietary patterns with traditional foods such as Tibetan salt cream tea, Zanba, and Tibetan milk tea being important daily foods. Tibetan foods were the main sources of intakes of iron and zinc. Intakes of these nutrients were higher than nutrient reference values and intakes reported in the 2002 NNHS. Nutritional education could be considered as one potential measure for improving the dietary patterns among this population.

## Competing interests

The authors declare that they have no competing interests.

## Authors' contributions

ZW was involved in the preparation of the research protocol, field management, collection and analysis of data, and manuscript writing. HY and SD were responsible for the study design and sampling, field management, and preparation of protocols. All authors read and approved the final manuscript.

## Pre-publication history

The pre-publication history for this paper can be accessed here:

http://www.biomedcentral.com/1471-2458/10/801/prepub

## References

[B1] Yin S Chinese Women's Nutrition and Health Status (Childbearing-age, Pregnancy, Breast-feeding) - 2002 National Nutrition and Health Investigation in China 2008 Beijing: People Health Press

[B2] Xing Y Yan H Dang S Zhuoma B Zhou X Wang D Hemoglobin levels and anemia evaluation during pregnancy in the highlands of Tibet: a hospital-based study BMC Public Health 2009 15 336 10.1186/1471-2458-9-336 PMC2753353 19754927

[B3] Kimura Y Okumiya K Sakamoto R Ishine M Wada T Kosaka Y Wada C Ishimoto Y Hirosaki M Kasahara Y Konno A Chen W Otsuka K Fujisawa M Nakatsuka M Nakashima M Wang H Dai Q Yang A Gao J Li Z Qiao H Zhang Y Ge RL Matsubayashi K Comprehensive geriatric assessment of elderly highlanders in Qinghai, China IV: comparison of food diversity and its relation to health of Han and Tibetan elderly Geriatr Gerontol Int 2009 9 359 365 10.1111/j.1447-0594.2009.00543.x 20002755

[B4] Ge K Zhai F Wang Q Effect of nationality on dietary pattern and meal behavior in China Am J Clin Nutr 1997 65 1290S 1294S 9094935 10.1093/ajcn/65.4.1290S

[B5] Liu R China Population, Section of Tibet 1988 Beijing: Publishing House of Finance and Economy

[B6] National Bureau of Statistics of China China Statistics Yearbook 2004 Beijing: China Statistics Press

[B7] National Bureau of Statistics of Tibet Tibet Statistics Yearbook 2001 Beijing: China Statistics Press

[B8] Dang S Yan H Yamamoto S Wang X Zeng L Poor nutritional status of younger Tibetan children living at high altitudes Eur J Clin Nutr 2004 58 938 946 10.1038/sj.ejcn.1601915 15164115

[B9] Dang S Yan H Yamamoto S Wang X Zeng L Feeding practice among younger Tibetan children living at high altitudes Eur J Clin Nutr 2005 59 1022 1029 10.1038/sj.ejcn.1602207 15970940

[B10] Clinical guidelines on the identification, evaluation, and treatment of overweight and obesity in adults WMJ 1998 97 20 1 24-5, 27-37 9810255

[B11] Cheng Y Yan H Dibley MJ Shen Y Li Q Zeng L Validity and reproducibility of a semi-quantitative food frequency questionnaire for use among pregnant women in rural China Asia Pac J Clin Nutr 2008 17 166 177 18364342

[B12] Cheng Y Dibley MJ Zhang X Zeng L Yan H Assessment of dietary intake among pregnant women in a rural area of western China BMC: Public Health 2009 9 222 10.1186/1471-2458-9-222 19589154 PMC2716336

[B13] Venter CS MacIntyre UE Vorster HH The development and testing of a food portion photograph book for use in an African population J Hum Nutr Diet 2000 13 205 218 10.1046/j.1365-277x.2000.00228.x 12383127

[B14] Huybregts L Roberfroid D Lachat C Van Camp J Kolsteren P Validity of photographs for food portion estimation in a rural West African setting Public Health Nutr 2008 11 581 587 10.1017/S1368980007000870 17686204

[B15] Institute of Nutrition and Food Safety, China CDC China Food Composition 2002 2002 Beijing: Peking University Medical Press

[B16] Institute of Nutrition and Food Safety, China CDC China Food Composition 2004 2005 Beijing: Peking University Medical Press

[B17] Zhai F He Y Wang Z Hu Y Wang HJ Li Y Status and characteristics of dietary intake of 12 minority nationalities in China Wei Sheng Yan Jiu 2007 36 539 541 18095560

[B18] Hurrell R Egli I Iron bioavailability and dietary reference values Am J Clin Nutr 2010 91 1461S 1467S 10.3945/ajcn.2010.28674F 20200263

[B19] Carpenter CE Mahoney AW Contributions of heme and nonheme iron to human nutrition Crit Rev Food Sci Nutr 1992 31 333 367 10.1080/10408399209527576 1581009

[B20] Hunt JR Moving toward a plant-based diet: are iron and zinc at risk? Nutr Rev 2002 60 127 134 10.1301/00296640260093788 12030275

[B21] Thankachan P Muthayya S Walczyk T Kurpad AV Hurrell RF An analysis of the etiology of anemia and iron deficiency in young women of low socioeconomic status in Bangalore, India Food Nutr Bull 2007 28 328 336 17974366 10.1177/156482650702800309

[B22] Hunt JR Bioavailability of iron, zinc, and other trace minerals from vegetarian diets Am J Clin Nutr 2003 78 633S 639S 12936958 10.1093/ajcn/78.3.633S

[B23] Ruel MT Is dietary diversity an indicator of food security or dietary quality? A review of measurement issues and research needs Food Nutr Bull 2003 24 231 232 12891828 10.1177/156482650302400210

[B24] Torheim LE Ouattara F Diarra MM Thiam FD Barikmo I Hatløy A Oshaug A Nutrient adequacy and dietary diversity in rural Mali: association and determinants Eur J Clin Nutr 2004 58 594 604 10.1038/sj.ejcn.1601853 15042127

[B25] Johansson L Thelle DS Solvoll K Bjørneboe GE Drevon CA Healthy dietary habits in relation to social determinants and lifestyle factors Br J Nutr 1999 81 211 220 10.1017/S0007114599000409 10434847

[B26] Hjartaker A Lund E Relationship between dietary habits, age, lifestyle and socio-economic status among adult Norwegian women. The Norwegian Women and Cancer Study Eur J Clin Nutr 1998 52 565 572 10.1038/sj.ejcn.1600608 9725656

[B27] Hulshof KF Löwik MR Kok FJ Wedel M Brants HA Hermus RJ ten Hoor F Diet and life style factors in high and low socio-economic groups (Dutch Nutrition Surveillance System) Eur J Clin Nutr 1991 45 441 450 1959516

[B28] Smith AM Baghurst KI Public health implications of dietary differences between social status and occupational category groups J Epidemiol Community Health 1992 46 409 416 10.1136/jech.46.4.409 1431718 PMC1059611

[B29] Smith AM Smith C Dietary intake and lifestyle patterns: correlates with socio-economic, demographic and environmental factors Hum Nutr Diet 1994 7 283 294 10.1111/j.1365-277X.1994.tb00271.x

[B30] Giskes K Turrell G Patterson C Newman B Socio-economic differences in fruit and vegetable consumption among Australian adolescents and adults Public Health Nutr 2002 5 663 669 10.1079/PHN2002339 12372160

[B31] Shahar D Shai I Vardi H Shahar A Fraser D Diet and eating habits in high and low socioeconomic groups Nutrition 2005 21 559 566 10.1016/j.nut.2004.09.018 15850961

[B32] Irala-Estevez JD Groth M Johansson L A systematic review of socioeconomic differences in food habits in Europe: consumption of fruit and vegetables Eur J Clin Nutr 2000 54 706 714 10.1038/sj.ejcn.1601080 11002383

[B33] Morgenstern H Thomas D Principles of study design in environmental epidemiology Environ Health Perspect 1993 101 23 38 10.2307/3431657 8206038 PMC1519688

